# The Chondroprotective Role of TMF in PGE_2_-Induced Apoptosis Associating with Endoplasmic Reticulum Stress

**DOI:** 10.1155/2015/297423

**Published:** 2015-09-07

**Authors:** Jianqiong Yang, Haiqing Liu, Linfu Li, Hai Liu, Weimei Shi, Longhuo Wu

**Affiliations:** ^1^Department of Clinical Research Center, The First Affiliated Hospital of Gannan Medical University, Ganzhou 341000, China; ^2^College of Pharmacy, Gannan Medical University, Ganzhou 341000, China

## Abstract

Endoplasmic reticulum stress (ERS) has been demonstrated to exhibit a critical role in osteoarthritic chondrocytes. Whether 5,7,3′,4′-tetramethoxyflavone (TMF) plays the chondroprotective role in inhibition of PGE_2_-induced chondrocytes apoptosis associating with ERS has not been reported. To investigate this, the activation of PERK, ATF6, and IRE1 signaling pathways in ERS in chondrocytes pretreated with PGE_2_ was studied. By treatment with PGE_2_, the chondrocytes apoptosis was significantly increased, the proapoptotic CHOP and JNK were upregulated, the prosurvival GRP78 and XBP1 were downregulated, and GSK-3*β* was also upregulated. However, TMF exhibited the effectively protective functions via counteracting these detrimental effects of PGE_2_. Finally, the inflammatory cytokine PGE_2_ can activate ERS signaling and promote chondrocytes apoptosis, which might be associated with upregulation of GSK-3*β*. TMF exhibits a chondroprotective role in inhibiting PGE_2_-induced ERS and GSK-3*β*.

## 1. Introduction

Endoplasmic reticulum stress (ERS) might be stimulated by a dysfunctional activity of ER dealing with the misfolded or unfold proteins [1]. The resident transmembrane proteins (PERK, ATF6, and IRE1) are the three ER sensors involved in unfolded protein response (UPR), which functions to maintain the homeostasis of ER by attenuating protein translation, reducing the load of newly synthesized protein, degrading misfolded or unfolded protein, and upregulating chaperones, such as Grp78 [2]. However, if the protective responses fail and the detrimental factors and ERS persist, cells are indexed to switch from prosurvival to proapoptosis [3], leading to enhanced expression of CHOP and upregulation of ER-related caspase-12.

Recently, it has been demonstrated that ERS is sensitively implicated in chondrocytes and associated with human osteoarthritis cartilage [4]. PERK and ATF6 signaling pathways activate the downstream factor CHOP to downregulate the expression of Bcl-2, an antiapoptotic protein, and contribute to programmed cell death [5], which has been comprehensively reviewed [6]. However, XBP1 and the chaperone GRP78 exhibit prosurvival functions to promote protein processing and provision during ERS. GSK-3*β*, which is a multifunctional Ser/Thr kinase, has been identified to be a major regulator of inflammatory responses and be involved in ERS [7]. GSK-3*β* has been showed to regulate ERS-activated CHOP expression. Inhibition of GSK-3*β* can significantly downregulate the expression of CHOP in neuronal cells [8]. In our previous studies, we found that PGE_2_ could upregulate the expression of GSK-3*β* and this could be significantly inhibited by 5,7,3′,4′-tetramethoxyflavone (TMF), which is one of active polymethoxyflavone compounds isolated from* Murraya exotica* [9]. In this paper, we will discuss whether PGE_2_ can induce activation of ERS and GSK-3*β* in chondrocytes and the protection role of TMF.

## 2. Materials and Methods

### 2.1. Materials

TMF has been isolated from the leaves of* M. exotica* and purified by repeated column chromatography and recrystallization in acetoacetate. The purity was found to be more than 98%. Fetal bovine serum (FBS), Dulbecco's modified Eagle's minimum essential medium (DMEM) (low glucose), penicillin, and streptomycin were purchased from Gibco (Life Technologies, NY, USA). GRP78, PERK, p-PERK, eIF2*α*, p-eIF2*α*, ATF6, IRE1*α*, XBP1s, CHOP, JNK, GSK-3*β*, Bax, Bak, and GAPDH monoclonal antibodies and the peroxidase-conjugated secondary antibody were obtained from Abcam (Cambridge, UK).

### 2.2. Primary Cell Cultures

The ethical agreement has been granted by the Institutional Animal Care and Use Committee of Gannan Medical University. The cartilage was obtained from the knee joint of four-week-old rats under sterile conditions. In order to remove other tissues and cells, 0.25% pancreatic enzymes have been used for digestion of the cartilage for 30 min. Then, 0.2% collagenase II was used to continue to digest at 37°C for 4 h. Cells were cultured at 37°C with 5% CO_2_ in 10% FBS of the medium, including DMEM (low glucose), 100 U/mL penicillin, and 100 mg/mL streptomycin. The primary chondrocytes were used for the assays.

### 2.3. Tunnel and DAPI Staining Assay

Chondrocytes were cultured and administrated with 1 *μ*M PGE_2_ and TMF. After 48 h treatment, cells were harvested. 4% paraformaldehyde was employed to fix the cells. A tunnel and DAPI staining kit (Abcam, USA) was used for determining the cell apoptosis. A Leica DM3000 microscope was employed for all fluorescent images. And DFC 420 camera (Leica, Germany) functions to take the photos.

### 2.4. Quantitative Analysis of Apoptosis Cells

The FITC-annexin V/PI double-fluorescence labeling kit was used for analyzing the cell apoptosis by flow cytometry. The procedures were strictly performed under the recommendation of the kit (Nanjing KeyGEN Biological Technology Development Co., Ltd., Nanjing, China). After treatment with 1 *μ*M PGE_2_ and TMF, cells (1 × 10^6^/mL) were centrifuged and incubated to be stained by FITC-annexin V and PI. The flow cytometer (FACSCalibur BD, San Jose, CA) was employed for measurement of apoptosis.

### 2.5. Gene Expression Analysis

The easy-spin total RNA extraction kit (iNtRON Biotechnology, Seoul, Korea) was used for extraction of the total RNA from chondrocytes. Following standard protocols, M-MLV (Promega, USA) was employed to reverse-transcribe total RNA to cDNA, that is, using 2 *μ*g of total RNA to synthesize the first strand of cDNA for each sample. EzOmics SYBR qPCR kits purchased from Biomics in a Mastercycler (Eppendorf) were used for detection of the genes expression of* CHOP*,* GRP78*,* XBP1*,* Bax*, and* Bak*. The primer sequences were listed in Table 1. Amplification procedure was 94°C for 5 min, followed by 30 cycles at 94°C for 30 s, 56°C for 45 s, 72°C for 45 s, and finally 72°C for 10 min. The procedures of PCR assay were made by employing the iCycler iQ real time PCR system (Bio-Rad).

The PCR assays were set to perform with four duplications.* GAPDH* was used as an internal standard control. Primer and template designs following the same criteria for each target, primers, and Mg^2+^ concentrations had been optimized to render efficiency for each target near one per assumption underlying the 2^−ΔΔCT^ method [15].

### 2.6. Western Blot Analysis

After lysis in the buffer (2% SDS, 10% glycerol, 10 mmol/L Tris, pH 6.8, and 100 mmol/L DTT), cells were performed with immunoblotting. Using bovine serum albumin as a standard, the protein concentrations were determined by employing a BCA Protein Assay Kit (Pierce Biotechnology, Rockford, IL, USA). After being mixed with gel loading buffer (50 mmol/L Tris-HCl, pH 6.8, 2% SDS, 10% glycerol, and 0.1% bromophenol blue) and denatured for 5 min, each sample (50 *µ*g) was electrophoresed on 10% SDS-PAGE gel for GRP78, PERK, p-PERK, eIF2*α*, p-eIF2*α*, ATF6, IRE1*α*, XBP1s, CHOP, JNK, GSK-3*β*, Bax, and Bak, respectively. Proteins were separated and Western-blotted onto a transfer membranes made of polyvinylidene difluoride (PVDF). Tris-buffered saline (TBS) containing 5% nonfat milk was used to block the blots for 1 h. Then, these blots were incubated with GRP78, PERK, p-PERK, eIF2*α*, p-eIF2*α*, ATF6, IRE1*α*, XBP1s, CHOP, JNK, GSK-3*β*, Bax, and Bak at 4°C over night, respectively. Rinsed by PBS, these blots were then incubated with HRP-conjugated goat anti-rat IgG for 1 h. After washing, the blots were set for detection. A super enhanced chemiluminescence detection kit (Applygen Technologies Inc., Beijing, China) was employed, and the protein bands were visualized after exposure of the membranes to Kodak film (USA). GAPDH was used as the internal control in all Western blot analyses.

### 2.7. Statistical Analysis

All data were presented as mean ± standard deviation (SD). Gene expression data were statistically analyzed by a paired *t*-test. Differences were considered significant at *p* < 0.05.

## 3. Results

### 3.1. TMF Decreased PGE_2_-Induced Apoptosis Ratio of Chondrocytes In Vitro

**Figure 1:**
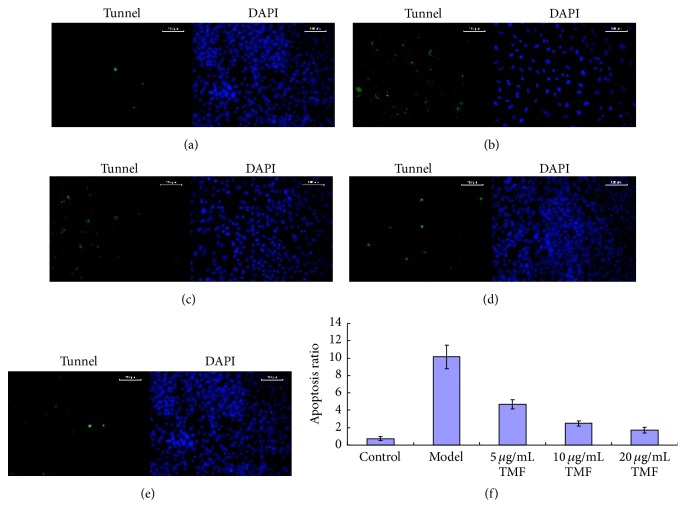
TMF inhibited chondrocytes apoptosis induced by 1 *μ*M PGE_2_. The normal chondrocytes were incubated for 48 h. In the control group (a), chondrocytes were incubated with no medicines. Model group (b) was the normal chondrocytes incubated with 1 *μ*M PGE_2_. Figures (c)–(e) were the groups incubated with 1 *μ*M PGE_2_ and 5, 10, and 20 *μ*g/mL TMF, respectively. Figure (f) was the summarized data obtained from tunnel staining to indicate the rate of apoptosis cells. Data were expressed by mean ± SD of 4 replicates. ^*∗*^ *p* < 0.05 as compared with control.

**Figure 2:**
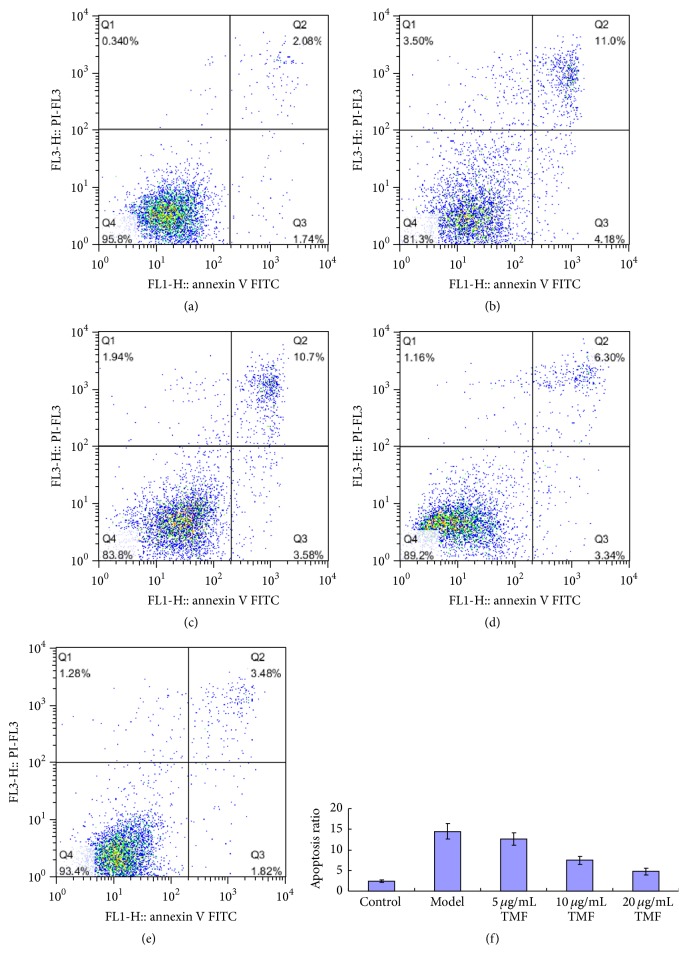
TMF inhibited chondrocytes apoptosis induced by 1 *μ*M PGE_2_. The normal chondrocytes were incubated for 48 h. In the control group (a), chondrocytes were incubated with no medicines. Model group (b) was the normal chondrocytes incubated with 1 *μ*M PGE_2_. Figures (c)–(e) were the groups incubated with 1 *μ*M PGE_2_ and 5, 10, and 20 *μ*g/mL TMF, respectively. Figure (f) was the summarized data obtained from flow cytometry to indicate the rate of apoptosis cells. Data were expressed by mean ± SD of 4 replicates. ^*∗*^ *p* < 0.05 as compared with control.

To observe the effect of TMF on chondrocytes cell death, 1 *μ*M PGE_2_ and TMF (5, 10, and 20 *μ*g/mL) were added to the cultured medium for 48 h. Cell apoptosis was analyzed by tunnel staining and flow cytometry using the FITC-annexin V/PI double staining. As showed in Figures 1 and 2, PGE_2_ could significantly promote chondrocytes apoptosis, and TMF exhibited effectively chondroprotective activity. In the model group, the chondrocytes apoptosis rates were 10.14 ± 1.36% in tunnel staining (Figure 1) and 14.50 ± 1.89% in FITC-annexin V/PI double staining (Figure 2). In contrast, at the dose of 20 *μ*g/mL of TMF, they showed that the chondrocytes apoptosis rates are almost as moderate as those in the control group.

### 3.2. TMF Downregulated the Expressions of* CHOP*,* Bax*, and* Bak* but Upregulated* GRP78* and* XBP1* Genes

**Figure 3:**
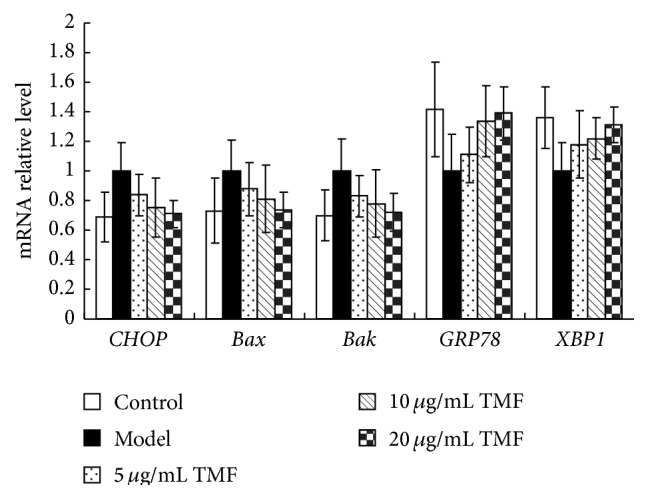
Changes in* CHOP*,* Bax*,* Bak*,* GRP78*, and* XBP1* mRNA expression. Chondrocytes in the control, the model group, and treated groups were incubated with 1 *μ*M PGE_2_ and 5, 10, and 20 *μ*g/mL TMF for 48 h, respectively. The changes of mRNA expression of these genes were detected by qRT-PCR. GAPDH was used as internal control. Data were presented by mean ± SD of 4 replicates. ^*∗*^ *p* < 0.05 as compared with the model.

PGE_2_, produced by COX-2, is a proinflammatory cytokine. To determine whether PGE_2_ exhibits its inflammatory role in inducing ERS, changes in* CHOP*,* Bax*,* Bak*,* GRP78*, and* XBP1* mRNA expressions were assessed using qRT-PCR (Figure 3). It showed that PGE_2_ upregulated the proapoptotic gene expressions of* CHOP*,* Bax*, and* Bak* but downregulated prosurvival genes* GRP78* and* XBP1*. By treatment with TMF for 48 h, the mRNA expressions of* CHOP*,* Bax*,* Bak*,* GRP78*, and* XBP1* showed an opposite difference from those in model groups and exhibited chondroprotective activity in a dose-dependent manner.

### 3.3. TMF Ameliorated the Protein Expressions in ERS Signaling and Downregulated the Protein Expression of GSK-3*β*

**Figure 4:**
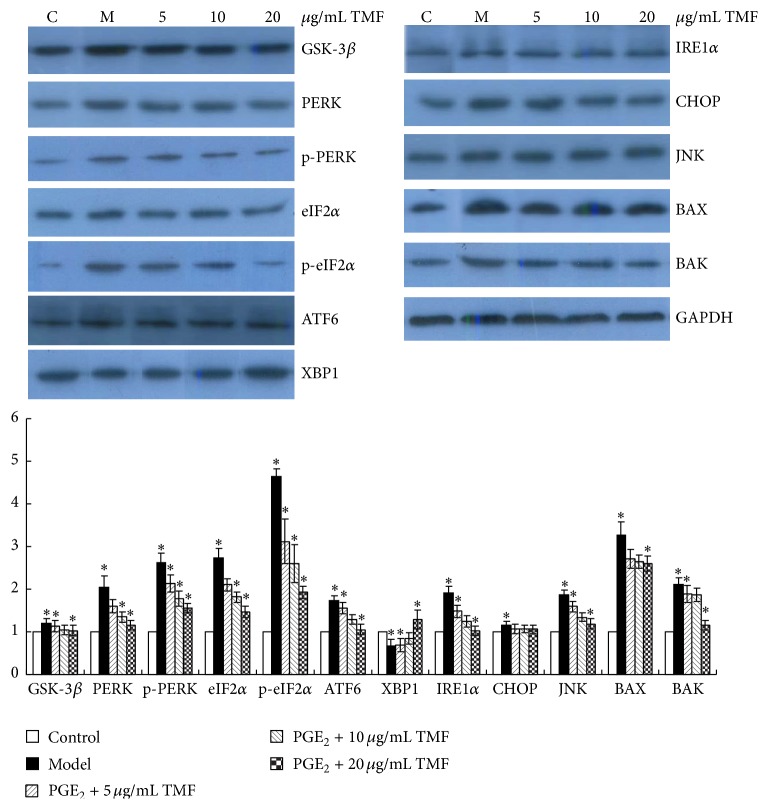
Changes in protein expression of GSK-3*β*, PERK, p-PERK, eIF2*α*, p-eIF2*α*, ATF6, IRE1*α*, XBP1, CHOP, BAX, and BAK. Chondrocytes in the control, the model groups, and the treated groups were incubated with 1 *μ*M PGE_2_ and 5, 10, and 20 *μ*g/mL TMF for 48 h, respectively. The changes of proteins expression were detected by Western blot. GAPDH was used as internal control. Data were presented by mean ± SD of 4 replicates. ^*∗*^ *p* < 0.05 as compared with the model.

We used PGE_2_ to further determine whether it ameliorated the protein expressions in ERS signaling and downregulated the protein expression of GSK-3*β*. The chondrocytes cells were incubated with 1 *μ*M PGE_2_ and different doses of TMF for 48 h and then harvested to be lysed to prepare for Western blot assays. The data in Figure 4 revealed that PGE_2_ significantly upregulated the protein expressions of PERK, ATF6, and IRE1 signaling but downregulated the expression of XBP1. By treatment of chondrocytes with TMF, the protein levels of PERK, p-PERK, eIF2*α*, p-eIF2*α*, ATF6, IRE1*α*, CHOP, BAX, and BAK were significantly downregulated dose-dependently, as compared with those in the model group. But the expression of XBP1 was upregulated. We also found that the protein expression of GSK-3*β* was activated by PGE_2_ and downregulated by TMF. This might indicate that GSK-3*β* was involved in regulating of ERS signaling partly.

## 4. Discussion

Apoptosis has been implicated in the progression of chronic degenerative conditions, such as osteoarthritis. Chondrocytes, the unique cell type in cartilage, are implicated in a multiple of stressors, including biomechanical, oxidative, and inflammatory stress [4], and mechanism must be available for maintaining balance regardless of these stressors. ERS signaling pathway has been reported to contribute to several human diseases, including osteoarthritis [16]. In the current study, we investigated PGE_2_-induced apoptosis associating with ERS in chondrocytes and the chondroprotective role of TMF in downregulation of ERS signaling pathway.

Firstly, we investigated that chondrocytes apoptosis induced by tunicamycin (TM) was increased (S1), which could be inhibited by administration of TMF. This confirmed that TMF could be a potential inhibitor of ERS to protect chondrocytes. Induction of CHOP is considered as a critical event for ERS-mediated apoptosis. CHOP has been demonstrated to directly contribute to apoptosis induced by cytokines by activating mitochondrial apoptosis pathways in *β*-cells [17]. In accordance with this, PGE_2_ could significantly upregulate the expression of CHOP in mRNA and protein levels, which also indicated activation of ERS in chondrocyte cells model. The UPR can be regulated by the activation of ATF4 and ATF6, which are two transcription factors that could activate the expression of* CHOP*. Released from GRP78, activated phosphorylated PERK phosphorylates eIF2*α*, which downregulates the translation via inhibiting the activity of the guanine nucleotide exchange factor eIF2B and blocking of eIF2-GDP recycled to eIF2-GTP [18]. However, ATF4 is activated to induce stress responsive genes, such as proapoptotic* CHOP* [19]. Similar to PERK, active ATF6 is translocated to the Golgi to be cleaved. The cleavage is then translocated to the nucleus to promote the stress responsive genes expression [20]. By treatment with TMF, the expressions of PERK, p-PERK, eIF2*α*, p-eIF2*α*, and ATF6 were significantly downregulated.

On the other hand, GRP78, an endoplasmic reticulum chaperone, plays an important protective role in protein processing and provision in UPR [21]. IRE1*α* possesses both kinase and ribonuclease activity. IRE1*α* is inactivated by binding to GRP78. Activation of ERS promotes the release of IRE1 from GRP78, leading to oligomerizaion, autophosphorylation, and activation of IRE1. The mRNA of* XBP1* is spliced by the active IRE1a to produce and accumulate the active XBP1, which upregulates the expression of chaperones, such as GRP78 [20]. Active XBP1 exerts its prosurvival effect under conditions of ERS through interacting with Hsp72, which is reported to inhibit the activities of CHOP and JNK [22]. Our findings revealed that the mRNA expressions of* GRP78* and* XBP1* were significantly upregulated, after treatment for 48 h with TMF. IRE1*α* activation has been reported to interact with tumor necrosis factor receptor-associated factor 2 (TRAF2) to promote a cascade of phosphorylation events, including activation of the MAP3K apoptosis signal-regulated kinase 1 (ASK1) and JNK [23], which is an important factor in pro- and antiapoptotic events in the cells. The BH3-only proteins BID and BIM can be phosphorylated by JNK to exhibit their proapoptotic roles involved in the activation of BAX and BAK, which are found to directly interact with IRE1*α* to form a protein complex and to regulate its activity [24]. Stimulated by PGE_2_, the expressions of JNK, BAX, and BAK were significantly upregulated but decreased with treatment with TMF for 48 h.

GSK-3*β*, a ubiquitously expressed Ser/Thr kinase, has a broad range of substrates and is involved in cell activation, differentiation, and survival [25]. It has been demonstrated that GSK-3*β* plays an important positive role in the inflammatory process [26]. Also, GSK-3*β* has been associated with ERS-induced apoptosis. Studies show that GSK-3*β* inhibitors reduce the apoptosis induced by ERS, and valproate (2-propylpentanoic acid), via inhibiting the activity of GSK-3*β*, inhibits ERS-induced apoptosis [27]. By treatment with GSK-3*β* siRNA, the apoptosis induced by ERS stimulants thapsigargin (TG) and TM can be abrogated [28]. Similarly, in PC12 cells and HepG2 cell, GSK-3*β* inhibitors can also significantly attenuate TG-induced apoptosis [29]. Our findings showed that the inflammatory cytokine PGE_2_-induced upregulation of GSK-3*β* might be associated with progression of ERS and chondrocytes apoptosis, which should be further investigated. These would be inhibited by administration of TMF, which is rich in Chinese traditional medicine and fruits and became a potential drug for chondrocytes protection.

In summary, the inflammatory cytokine PGE_2_ can activate ERS signaling and promote chondrocytes apoptosis, which might be associated with upregulation of GSK-3*β*. TMF exhibits a chondroprotective role in inhibiting PGE_2_-induced ERS and GSK-3*β*.

## Supplementary Material

TMF decreased chondrocytes apoptosis induced by TM.

## Figures and Tables

**Table 1:** Primer sequences for different genes.

*CHOP*	Forward Reverse	5′-CAGAACCAGCAGAGGTCACA-3′ 5′-AGCTGTGCCACTTTCCTTTC-3′	Reference [10]

*GRP78*	Forward Reverse	5′-GCTCGACTCGAATTCCAAAG-3′ 5′-TTTGTCAGGGGTCTTTCACC-3′	Reference [11]

*XBP1*	Forward Reverse	5′-AGTTAAGAACACGCTTGGGAAT-3′ 5′-AAGATGTTCTGGGGAGGTGAC-3′	Reference [12]

*Bax*	Forward Reverse	5′-TCCCCCCGAGAGGTCTTTT-3′ 5′-CGGCCCCAGTTGAAGTTG-3′	Reference [13]

*Bak*	Forward Reverse	5′-GTCCGGACGCACTTGAAGAACTT-3′ 5′-AGCATCAATTTCATCGCCTATCTCT-3′	Reference [14]

*GAPDH*	Forward Reverse	5′-CAGTGGCAAAGTGGAGATTG-3′ 5′-AATTTGCCG-TGAGTGGAGTC-3′	Reference [15]
