# Supplementary material for: The Chondroprotective Role of TMF in PGE2-Induced Apoptosis Associating with Endoplasmic Reticulum Stress
Source: Evid Based Complement Alternat Med. 2015 Sep 7;2015:297423. doi: 10.1155/2015/297423 (PMC4576019; doi:10.1155/2015/297423)
Supplement: Supplementary file 1 — TMF decreased chondrocytes apoptosis induced by TM. [file 297423.f1.pdf]

Supplement

S1: TMF decreased TM-induced apoptosis ratio of chondrocytes in vitro

To determine the effect of TMF on chondrocytes cell death, 1 μM TM and TMF (5, 10, and 20 μg/mL) were added to the cultured medium for 48 h. Cell apoptosis was analyzed by flow cytometry using the FITC-Annexin V/PI double staining. As showed in Figure S1, TM could promote chondrocytes apoptosis, and TMF exhibited chondroprotective activity. In the model group, the chondrocytes apoptosis rates were 4.38 ± 0.34% in FITC-Annexin V/PI double staining. In contrast, at the dose of 20 μg/mL of TMF, they showed almost as moderate as those in the control group.

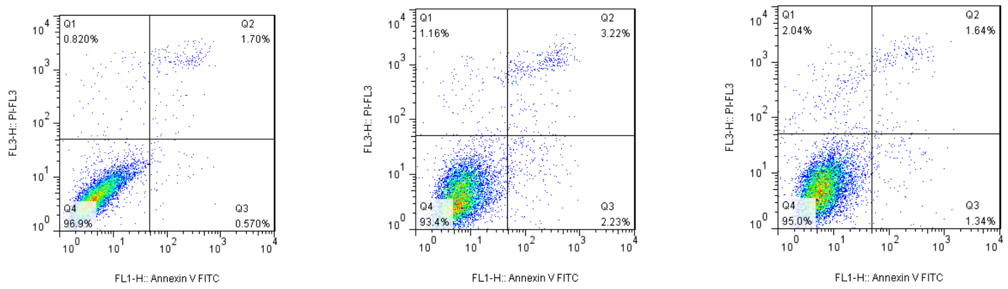

A B C

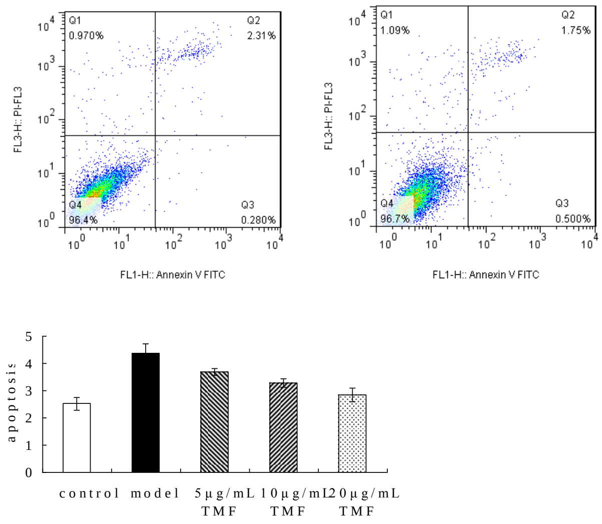

D E F

Figure S1. TMF inhibited chondrocytes apoptosis induced by 1 μM TM. The healthy chondrocytes were incubated for 48 h. Chondrocytes in the control group (A) was incubated without adding any medicines. Model group (B) was the healthy chondrocytes incubated with 1 μM TM. Figure C-E were groups incubated with 1 μM TM and 5, 10, and 20 μg/mL TMF, respectively. Figure F was the summarized data indicating the rate of apoptosis cells, as detected by flow cytometry. Data were presented by mean ± standard deviation of 4 replicates. *p < 0.05 as compared with control.
